# Manual of Morbid Anatomy

**Published:** 1843-01

**Authors:** 


### ART. VII.

1. *Handboek der Ziektekundige Ontleedkunde.* Door W. VROLIK, Med. Doctor, Hoogleerar aan het Athenæum te Amsterdam, enz. Eerste Deel. *Aangeborene Gebreken.—Amsterdam,* 1840. 8vo, pp. 590.

*Manual of Morbid Anatomy.* By W. VROLIK, M.D., Professor at the Athenæum of Amsterdam, &c. Vol. I. *Congenital Diseases.—Amsterdam,* 1840.

2. *Specielle pathologische Anatomie.* Von Dr. KARL EWALD HASSE, ausserordentlichem Professor der Medicin zu Leipzig. Erster Band. —*Leipzig,* 1841. 8vo, pp. 526.

*Special Pathological Anatomy.* By Dr. K. E. HASSE, Professor Extraordinary of Medicine at Leipzig. Vol. I.—*Leipzig,* 1841.

3. *Handbuch der Pathologischen Anatomie.* Von CARL ROKITANSKY, Med. Dr. Custos der k. k. pathol. Museums, und a.ö. Professor zu Wien. III. Band.—*Wien,* 1842. 8vo, pp. 632.

*Manual of Morbid Anatomy.* By CARL ROKITANSKY, M.D., Conservator of the Royal Imperial Pathological Museum and Professor extraordinary, at Vienna. Vol. III.—*Vienna,* 1842.

WE have here the first published volumes of three systems of morbid anatomy, each important not only by the extent of the plan on which it is commenced, but by the reputation of its author. Dr. Vrolik (the worthy son of a veteran anatomist, who, though he long ago earned an European reputation, still labours diligently in medical science,) is already well known by extended and successful investigations in both comparative and morbid anatomy, in the knowledge of which he is second to none in Holland. In the present work he proposes to bring into a systematic form all the knowledge which he has acquired of morbid anatomy, whether from the works of others, or from the rich stores of facts contained in his own and other Dutch museums; stores such as no other country in Europe can boast of. In its plan, his work is more complete than any hitherto published. Of this first volume, half is occupied by a clear and accurate account of all the circumstances of the normal development of the embryo, and of each of its several systems and organs. The second division relates to the deviations of development in general, including all the varieties of extra-uterine pregnancy, and the diseases of the ovum. The third is occupied by the congenital defects due to arrest or hindrance of development, and includes the descriptions of fissures or permanent openness of parts, hydrocephalus, and hydrorachitis, and the acephalous monsters. On all these subjects it is, so far as our present knowledge goes, complete. The second volume will conclude the account of congenital diseases; the third and fourth will be devoted to diseases after birth. Judging by the examples before us the work when finished will deserve to be the text-book of all to whom it is accessible; but who will translate it? At some future time we shall analyse the parts relating to monsters, and supply much of its interest to the general reader; but all its value will, we fear, long remain unknown to those who will not learn Dutch that they may read it.

Dr. Hasse's work is one not on morbid anatomy merely, but on pathology; for in each case the author discusses the general features of the history of the disease, as well as its signs after death. It is an admirable compendium of the writings of preceding authors, and is really beautifully written. It contains little of original observation, and not many new interpretations of facts; but for the student, or for one not far advanced in the knowledge of pathology, it is admirably adapted. This volume includes the diseases of the organs of respiration and circulation.

Unlike the two preceding, Dr. Rokitansky's book is no more than it professes to be; it is morbid anatomy in its densest and most compact form; scarcely ever alleviated by cases, histories, or hypotheses. It is just such a work as might be expected from its author, who is said to have written in it the results of his experience gained in the careful examination of 12,000 bodies, and who is possessed of a truly marvellous power of observing and amassing facts. But at present we need say no more; for in the following pages we purpose giving an analysis of the best contents of this volume, with occasional illustrations from that of Hasse, and with some comments. In this we hope to render some service to our readers, for, valuable as it is, there is not much probability that Rokitansky's work will be translated, and it is written in such vile crabbed Bohemian-German that few even of those to whom good German is familiar will ever wade through it.

The present volume will be, when the work is completed, the third and last. The first will relate to general morbid anatomy; the second to the morbid anatomy of the nervous, vascular, and motor systems; this contains the descriptions of the diseases of the respiratory, digestive, urinary and genital organs. In the mode of arrangement there is nothing striking; our notices may therefore follow the order of the work itself.

AFFECTIONS OF THE TRACHEA AND BRONCHI. A change in the trachea is described which will be recognized by many, and which Rokitansky regards as the result of repeated and chronic attacks of tracheal catarrh.

*Dilatation of the trachea.* "It is a dilatation of the posterior wall of the trachea, proceeding from hypertrophy and relaxation, with or without a hernial extrusion of the mucous membrane. . . . . The posterior wall of the trachea is relaxed, and presents an increase of surface which is especially obvious in the transverse direction. The mucous membrane, the transverse muscular fibres behind it, and the mucous glands, are at the same time increased in volume, and the gland-ducts are dilated; while the elastic, yellow, longitudinal fibres are attenuated and wasted. If the extrusion of the mucous membrane now take place, it passes gradually between the thickened transverse muscular bands, in the form of a fissure, or a funnel, and, at last, of a saccular recess, which is placed transversely, and is for the most part deepest near the ends of the tracheal rings. . . . . The larger this hernia or diverticulum is, the more prominently do the muscular fasciculi that limit it, stand out upon the inner surface of the trachea; upon which, when the herniæ are numerous, and follow closely upon one another, they form a lattice-work of transverse bands. . . . Such dilatations sometimes extend through the whole trachea, and even beyond it to the bronchi." (p. 3.)

*Dilatation of the Bronchi.* After describing the generally known uniform and saccular dilatations of the bronchi, Rokitansky points out

"A particular variety, the saccular dilatation of the extremities of the bronchi. These are often seen in the form of thinly-membraned vesicles filled tensely with air, which are set singly, or in groups, in the neighbourhood of the cicatrix-like contractions at the apices of the lungs after the extinction of tuberculous disease. One or more bronchial tubes compressed by the shrunken pulmonary parenchyma, and at last obliterated, pass through the substance of the lung (where it is impermeable by air and full of pigment, and of obsolete and calcified tubercles), and at the periphery expand into sacs which, according as the tube is obliterated or merely compressed, either resist pressure or may be slowly emptied." (p. 5.)

Of the origin of ordinary bronchial dilatation he adopts a view nearly allied to that of Dr. Corrigan. He believes that the dilatation is a secondary affection; that the primary change is a shrivelling and shrinking up of some portion of the parenchyma in consequence of bronchitis in the terminal branches; and that there is thus produced a tendency to a vacuum which is filled up by the stretching of one or more of the adjacent bronchial tubes. The chief positive evidence which he adduces for this view is the fact that there is always collapsed or wasted pulmonary tissue in the neighbourhood of a dilated bronchus; and that the degeneration of the former is always proportionate to the dilatation of the latter. But the explanation is, we think, unsound. When a part of a lung shrivels, there is no portion more capable of resisting the relatively exaggerated atmospheric pressure than the bronchial tubes, if their tissues be healthy. The pressure being equal upon every part of the air-tubes and cells, the latter must dilate long before healthy tubes; and hence it is that the shrinking of a part of a lung is so commonly followed by hypertrophy or vesicular emphysema of the adjacent parts. Dr. Corrigan's view is more reasonable inasmuch as he admits the additional influence of the contraction (comparable to that of the liver in cirrhosis,) of the tissue around the dilated bronchus. But, we believe that he also excludes too much the probability that the tubes themselves are diseased before their dilatation. The change is, doubtless, as Dr. Stokes has maintained, very analogous to the formation of aneurisms. By degeneration of its coats, (and, probably, in most instances, of the surrounding tissues also,) a part or the whole of a bronchial tube loses its elasticity, and it is dilated partially or throughout by an atmospheric pressure which it could before resist; just as an artery weakened by anormal deposit yields to an average pressure of the blood, and becomes dilated or aneurismal.

The mere contraction of tissue round a bronchial tube can never pull its walls asunder; the arterial walls are much more extensible, yet no contraction or shrivelling of the tissues round them ever produces dilatation or aneurism; for either of these to occur it is essential that the arterial walls themselves should first be weakened by disease.

*Tuberculous disease of the trachea and bronchi.* The account of tuberculous disease of the air-passages deserves particular notice. In the larynx, where he has never seen it occur without similar disease in the lungs, Rokitansky admits two primary forms of deposit; the grey granulation (miliary tubercle) in the submucous cellular tissue, and the yellow caseous tubercle infiltrated in the mucous membrane. Both pass through the ordinary progress of tuberculous disease; and both may, though they very rarely do, heal. Tuberculosis of the trachea is, he says, extremely rare; an assertion, the seeming strangeness of which he clearly explains in the next passage.

"One often finds," he says, "in laryngeal phthisis, little ulcers of the mucous membrane of the trachea, and they are frequently so numerous that they coalesce. They are shallow, and for the most part of an elongated, rounded, or strip-like form; they have slightly concave bases, are sometimes not discernible, except when the light falls obliquely upon them, appear raw or excoriated, pale or dark red, are uncovered, or covered only by a creamy diffluent exudation, and are surrounded by a livid redness, or a sharply-defined red areola. They are situated especially on the posterior wall of the trachea, often extend into the trunks of the bronchi, are sometimes strikingly predominant on the right or left side of the trachea or the corresponding branches, according as the right or left lung is chiefly diseased, and often occur at the same time in the fauces and on the mucous membrane of the mouth. But these [which will be at once recognized as corresponding to those usually regarded as tuberculous ulcers of the trachea] have nothing in common with the [real] tuberculous ulcer: they are the results of an exudative aphthous process, which is especially associated with the active laryngeal phthisis." (p. 37.)

Besides the common tuberculous affection of the bronchial tubes in the neighbourhood of cavities, or tuberculous infiltration, he describes a "primitive bronchial tuberculosis," occurring especially in children, and commonly accompanied by an intense tubercular disease of the bronchial glands.

"It is a disease of the terminal branches of the bronchi; at least it developes itself originally in them, and extends from them into the larger tubes. It occurs especially, like pulmonary tuberculosis (ordinary phthisis), in the upper lobes, but is contrasted with that disease, in that it is frequent in the peripheral or superficial ramifications, affects a larger section of the bronchial tree, and that, on a transverse section, one finds the pulmonary parenchyma traversed by large, thickly-walled, bronchial tubes, filled by caseous tuberculous matter. It is often combined with tuberculous infiltration of the pulmonary parenchyma, but often is completely independent. In the latter case the obstruction of the bronchial tubes leads to obliteration of the vesicles, and wasting of the parenchyma connected with them, and one then finds the obstructed tuberculous bronchi branching in a ligamentous, shrivelled, elastic, and tough tissue. The tuberculous matter, in these cases, passes through its usual changes. It either softens, and then the bronchial walls are not unfrequently destroyed, and involved in collections of tuberculous pus, collections in which (contrary to those which are far more frequently formed by softening of pulmonary tubercle) the destruction of the bronchi is the primary change; or else the tuberculous matter undergoes another, the calcareous, metamorphosis, which is especially apt to occur when the bronchus has been completely closed by it." (p. 38.)

This description is the more important because it affords, we are persuaded, a correct account of that tuberculous disease of the bronchi in which Dr. Carswell has, far too exclusively, made ordinary phthisis to consist; and of which others, with an equally unfounded exclusiveness, have denied the existence.

Affections of the Pleura. On the diseases of the pleura we find nothing striking in Rokitansky; but Hasse has two important observations. He has made a series of preparations of false membrane, by drying them, in conjunction with the adjacent portion of pleura, upon glass, in which state the blood-vessels and their contents are quite distinct.

"In all the cases," he says, "in which vessels had formed in the false membranes, they were immediate continuations of the branches ramifying in the serous membrane. These entered the false membrane at various parts, and branched in it partly in stars, partly in groups of tufted, nearly parallel, lines.... I have repeatedly placed portions of a similar gelatinous exudation which had been floating freely upon glass, to seek for independent vascular formations in them, but I have never succeeded in detecting a trace of vessels in them; so that I might fairly maintain, that if ever vessels are formed in such loose flocculi, they must have been at some former time fixed to the pleura, and subsequently detached from it." (p. 250.)

Hasse mentions also (p. 252), that where tuberculous matter has been effused with the ordinary products of pleuritis, it may be collected in an isolated mass, and undergo the calcareous metamorphosis, becoming converted into hard, earthy concretions, in the form of uneven, rough plates, which may sometimes be split into two layers, containing between them some of the remains of the caseous matter, and adhering tightly to both surfaces of the pleura. But we do not find any marks by which it can be decided that these are not ossifications of false membrane rather than calcified tubercles.

Affections of the Lungs. This division of the subject is full of important matter, more especially the work of Rokitansky.

*Emphysema of the lungs.* Rokitansky's description of the anatomical characters of this affection is exact. In his explanation of its causes he adopts a view somewhat similar to Laennec's, believing that it is commonly the result of forced inspirations, such as occur in croup, hooping-cough, &c. But he carries this opinion (which is probably true for a few cases) to the extent of ascribing the emphysema which is usually called spontaneous to a similar cause. It happens, he says, in persons who lead a sedentary life, and who therefore breathe but little with their diaphragm and abdominal muscles. These muscles, therefore, become weak and atrophied; and, for compensation, their possessors must inspire, though seldom, yet deeply and forcibly, with the other inspiratory muscles, and especially with those at the upper part of the chest; hence the greater frequency of emphysema at the upper and anterior parts of the lungs.

We think this explanation, though it be somewhat ingenious, very weak. Such emphysema occurs in active as well as in sedentary persons. The latter too needing, from their habits, less respiration, can afford to employ their diaphragm and abdominal muscles less than others, and that without needing compensation. Besides, to say nothing of he frequency of emphysema in horses, whose habits are the very opposite of sedentary, and of its greatest frequency in heavy, slowly-moving horses, whose respiration is most even, the fact that it is often hereditary proves that it is in great measure independent of accidental external causes, and that though these may have some influence in making its existence sooner or later obvious, yet that it is mainly due to an original weakness of the walls of the air-cells in parts or the whole of a lung. In consequence of this weakness the cells cannot, for the ordinary term of life, resist the average atmospheric pressure, and they are therefore gradually stretched. In certain cases a similar weakness of the cells is doubtless acquired by disease, producing some organic change, and destroying the elasticity of their walls, so that they yield to ordinary atmospheric pressure, and still more certainly to the force of repeated, sudden, and deep inspirations. The same diseases, also, probably produce those changes which are sometimes found in the tissue adjacent to the emphysema, and which Rokitansky, and many others, refer to the pressure of the emphysematous portions. But these can exercise no unusual pressure on the parts around them; the air-cells do not *grow* as they are distended, but are merely *stretched* by the atmospheric pressure, to which alone, therefore, the adjacent portions are subjected, as well after as they were before the production of emphysema.

*Pulmonary apoplexy.* Neither Rokitansky nor Hasse accept the opinion recently advanced, that pulmonary apoplexy is due to blood effused in the bronchial tubes and thence passing into the cells. The former adheres to Laennec's opinion, that in general the air-cells are its primary seat, and quotes in support of his view, 1st, that infarctus (compact apoplectic effusion,) has often been found when there has been no hemoptysis, which could scarcely fail to occur if the blood were poured into the bronchial tubes; and, 2dly, that pulmonary apoplexy is commonly connected with hypertrophy of the right ventricle, which could scarcely influence the bronchial arteries. We fully agree with him; but should point out obstruction at the left side of the heart as more important than the (usually coincident) hypertrophy of the right side; for the latter often exists without any tendency to pulmonary apoplexy, as, for instance, in emphysema of the lungs. We may add, too, that the non-occurrence of pulmonary apoplexy in emphysema is good evidence against its dependence on congestion of the bronchial blood-vessels; for in no disease are they more frequently gorged with blood.

*Pneumonia.* After a brief but comprehensive description of œdema of the lungs, Rokitansky proceeds to the important section on pneumonia, (p. 84,) of which he makes four varieties, namely, croupous, (ordinary or plastic pneumonia,) typhous, catarrhal, and interstitial. The following are some of the more remarkable passages.

The difficulty of distinguishing simple from inflammatory congestion of the lungs is admitted to be great. He lays down as the distinctive characters of the latter, the *colour* of the parenchyma, which approaches to a brownish red, and its *moistness*, both of which depend on a condition peculiar to pneumonia, namely, the filling of the tissue of the lung by blood which has just entered upon the inflammatory state, and which is brownish or brick-red, thin, but viscid, and mixed with dark grumous flocculi. Certainly no sign is so generally distinctive; but it may be added, that the best mode of detecting it is by examining the fluid which may be scraped from the cut surface of the lungs: in pneumonic congestion it always bears some resemblance to the rust-coloured sputa.

The seat of pneumonia Rokitansky holds to be the walls of the air-cells, that is, the pulmonary mucous membrane; so that it might be defined a "parenchymatous croup."

"The characteristic granulations are produced by the product of inflammation deposited in the cavities of the air-cells. Their formation, that is, the exudation, is preceded by the secretion of a sticky, tough, reddish-brown fluid into the cells, which produces the well-known *râle crepitant;* with the hepatization this diminishes, and the pulmonary cells are filled by plastic exudation. The granulation is at first roundish, dark red, rather hard and brittle, and appears, as it were, fused with the swollen dark red wall of the cell, and is difficult to isolate and extract. But as the inflammatory turgescence and the redness of the tissue moderate, the granulation itself becomes paler, reddish-gray, and at last yellowish-gray, its cohesion is diminished, and it swells up a little. A secretion of a glutinous mucus ensues around it, its connexion with the wall of the cell is rendered looser, it becomes more distinct, and appears to be inclosed by a bright red cell-wall, which makes it the more distinct the paler it grows. Lastly, it melts down into a puriform fluid, mixed with the glutinous mucus." (p. 90.)

The following summary of the arguments, in addition to actual observation, by which Rokitansky supports his view of the intra-cellular formation of the pneumonic granulations is, we think, complete and convincing:

"The granulation, regarded as a pulmonary cell swollen and obliterated by swelling, could not present either the anatomical relations or the metamorphoses which we deduce from it as a product of inflammation. The most considerable swelling of the pulmonary cells could not produce the volume of the hepatized lung; while our theory completely explains this fact. If the purulent infiltration were a suppuration of the interstitial tissue, a healing of this stage without abscess and breach of continuity would not be possible; but by means of partial expectoration, and partial absorption of the softened exudation, this takes place without any ulcerative destruction of the tissue, and the anatomical examination of a purulent pulmonary infiltration exhibits the cellular texture of the lung entirely uninjured. Lastly, the same process commonly goes on in the terminal branches of the bronchi as in the pulmonary cells." (p. 91.)

The account of the modifications which the pneumonic product presents in correspondence with the peculiarities in the state of the blood is clearly written; and the descriptions of the catarrhal and the interstitial pneumonia deserve especial notice, for the sake of the very probable explanations which they afford of two anomalous forms of the disease. The catarrhal* occurs very rarely in adults, less rarely in children:

"It is always lobular, always has a bronchitis of the tubes belonging to the diseased portion of the lung associated with it, and is a frequent accident of the various catarrhal diseases of childhood, especially of hooping-cough and *catarrhus suffocativus.* Its especial seat is in the superficial lobules, many of which are often affected, and which become blueish-red, dense, and moderately firm. The walls of the air-cells are swollen even to the closure of their cavities, which, when the swelling is less, contain a watery, mucous, and slightly frothy secretion. There is no trace of a granular texture discernible. The pulmonary substance around the diseased lobules being, for the most part, emphysematous, they appear (when they are situated at the surface,) depressed somewhat below the level, and are distinguished by their dark colour." (p. 107.)

* The name is unfortunately chosen, though the disease may consist in inflammation of isolated bronchi and their appended cells; for the same term is used by Hasse and many others for the old *peripneumonia notha,* to which it is just as well applicable.

The interstitial pneumonia is that which is very commonly described as chronic pneumonia:

"At first the tissue in the interstices of the lobules and between the smaller groups of cells appears (when there is not too much black pulmonary substance,) pale-reddish, and swollen by albuminous infiltration: the cells are either pale and, according to the degree of that swelling, more or less compressed; or else, when they participate in the inflammation they are reddened, and sometimes (though always finely) granulated. In course of time the material infiltrated in the interstitial tissue organizes itself, combines with it into a dense cellulo-fibrous substance, in which the cells are obliterated by the compression, and ultimately is converted into a homogeneous cellular tissue. One then finds whitish compact stripes, which sometimes creak under the knife, or shapeless masses of the same kind imbedded in the substance of the lung." (p. 107.)

This form, as its usual name implies, is generally chronic in its progress; it sometimes occurs spontaneously, and spreads from one lobule to another. It is most frequent at the apices of the lungs, and being often combined with a partial pleurisy, may at last assume all the characters of a cicatrix of the lung. But more commonly it is a consecutive affection occurring in the neighbourhood of apoplectic effusions, tuberculous collections, &c., around which it forms a kind of capsules. The new tissue which is produced commonly contains a large quantity of carbonaceous matter.

*Phthisis pulmonalis.* The section which Rokitansky has devoted to pulmonary phthisis is one of the best of the volume ; it is so good that, though the subject may be thought by some to have been long ago exhausted, it merits a careful abstract. Of tuberculous disease of the lungs he says, there are two distinct forms, namely, interstitial tubercular granulation, and tubercular infiltration or infiltrated tubercle. In the latter the morbid substance is effused in the air-cells themselves, in the former in their interstices.

The tuberculous infiltration is "hepatization by a tuberculous product." An ordinary croupous or plastic pneumonia deposits its usual product; and this, under the influence of a tuberculous diathesis, instead of being absorbed or becoming purulent, passes through various discolorations, and is metamorphosed into the yellow tubercle; in other words, it is tuberculized. The several stages from the fibrinous to the tubercular matter may be distinctly traced.

The infiltration may be *general*, or, as it is much more commonly, *lobular*, or *vesicular*, and this last is Bayle's pulmonary granulation. (?) It is, however, rarely a primary form ; but occurs usually in advanced stages of the granular tuberculosis. It indicates a phthisis which has run a tumultuous, acute course, with frequent attacks of pneumonia. It occurs especially in young subjects, and is always accompanied by enlargement and tuberculous disease of the bronchial glands.

The tubercular granulations may be deposited either singly or at greater or less distances, or in groups; and this *grouped* form is always to be distinguished from the *confluent* variety, in which all the single granulations are closely set together. The tubercular granulation, in any of these forms, appears first, either as the gray obscurely-transparent mass, of the size of a millet or hemp seed ; or, (as in many cases of acute tuberculosis,) as a granule smaller than a grain of sand, clear, transparent, and like a vesicle; or, in an intense degree of the tuberculous diathesis, it may be deposited at once as the yellow tubercle. In whichever form it occur, its outlines are never sharp, though they seem so ; for little processes may be traced from them into the surrounding tissue.

The granulations thus formed gradually coalesce, and this occurs most quickly when they are from the first arranged in groups. In this state they form a mass of any shape or size, completely overwhelming the pulmonary tissue, which can be traced in it only by its infiltrated black matter, and a few blood-vessels; but they are still quite distinct from tuberculous infiltration.

The tubercles in the lungs undergo the same peculiar metamorphoses as in other organs, passing through the state of softening to the formation of the cavity or vomica. Each discrete gray granulation softens from its centre, which becomes turbid, more opaque, and friable, and at last, fluid. The groups present similar softenings at the centre of each of their component tubercles. From the former results a small ulcer; from the latter, when all the tubercles have gone through the same process, a larger ulcer or cavity ; and Rokitansky dwells particularly on the mode in which these cavities enlarge.

The cavities thus formed, he says, spread by the successive changes of tuberculization, softening, breaking down, and removal of their walls in a regular eccentric progress; and when these go on rapidly the wall of the cavity consists of nothing but pulmonary tissue infiltrated with tubercle. As they approach, the cavities coalesce, and communicate by sinuses or apertures of various size, or all are laid into one.

But in a slower progress of the disease a more healthy inflammation is set up around the cavity. An albuminous, grayish-white, or reddish product is deposited, which closes, and ultimately produces a wasting of, the air-cells. It may be converted into a grayish or blackish layer of dense and tough cellular tissue; and it may be either persistent, or may have tubercles formed within or beneath it, and breaking through it. At the same time also with this effusion without the cavity, (which constitutes the *infiltration tuberculeuse gelatiniforme* of Laennec,) albumen is effused in a layer of soft false membrane within it. But this is probably repeatedly thrown off as tuberculous matter collects beneath it, breaks through it, and carries it away with the pus of the cavity ; and it may be assumed that in accordance with improvement or deterioration of the patient's health, and as the disease tends towards cure or towards increase, so either this albuminous product or tubercle is produced upon the walls of the cavity.

But in certain cases these albuminous effusions, which are always indications of curative processes, proceed to a proper cure. And they are not the only modes in which tuberculous disease may be brought to a favorable conclusion ; for in several distinct circumstances its progress is arrested. 1st. There may be a callous degeneration of the tissue around the cavity, or the formation of a membrane within it like a serous or a mucous membrane; the former being usually found when the disease is tranquil, the latter when there is much irritation. 2dly. The cavity may completely cicatrize, its walls gradually falling in and uniting, with obliteration of the bronchi, and sinking in of the surface of the lung, and perhaps of the wall of the chest also. 3dly. The cavity may, after partially shrinking,be filled by chalky matter from the metamorphosis of some remaining tubercle. 4thly. In the place of the cavity there may be produced a large callous mass of tissue, like that of cicatrices. Or, 5thly. The tubercle may not proceed to the formation of the cavity, but being arrested in its earlier progress, may diminish in size, and be changed into a gray or dirty-white mass of chalky matter, and at last into a hard concretion; changes which may ensue in either the granular or the infiltration form. And, lastly, at a still earlier stage, the tubercle being arrested in its progress may retrograde and become *obsolete*, shrivelling into an opaque, blueish-gray, cartilaginous knot, which is indisposed to any further metamorphosis.

Thus, in any stage of its progress the tuberculous disease may be arrested, and either removed or reduced to a state of inaction: and where, as is rarely the case, these changes occur in all the tuberculous matter that has been deposited, and the diathesis is wholly remedied, the cure of the disease is complete.

Such is Rokitansky's general account of the ordinary progress of pulmonary tuberculous disease, considered independently of its effects on adjacent tissues. It is in nearly every respect exactly accordant with our own observations, and is certainly both clearer and more complete than any yet published. His account of the accidents and associated phenomena of the disease is not less praiseworthy. He says rightly that only large bronchial tubes open into cavities, the small ones being closed by the secondary tuberculous deposits around and within them, and by the swelling of their mucous membrane. The openings into them, when recent, are always ulcerated, oblique, and abrupt; but when the wall of the cavity becomes callous they acquire a smooth edge of tough mucous membrane, which they retain permanently or till, as is rarely the case, they are obliterated. He points out tubercular infiltration as the most frequent precedent of perforation of the pleura; and this result is favoured by the frequency with which it occurs, especially at the surface of the lung, and the rapidity with which it is apt to break down and become fluid before adhesions are produced over it. In these, as well as in other cases of perforation, he well describes how the pleura is first distended by the air passing into the cavity till, having been raised like a small bladder on the surface of the lung, it bursts, or dies and is thrown off, or else sloughs, being involved with a small adjacent portion of the lung in gangrene.

Affections of the stomach and bowels. With some brief remarks on carcinoma of the lungs Rokitansky concludes his account of the morbid anatomy of the respiratory organs, and the best and most nearly complete section of the volume. In passing from it, as one does abruptly, to the account of the diseases of the upper part of the digestive canal, a striking instance is presented of the inequality in the merit of different parts of his work. His account of the affections of the lips, mouth, pharynx, and œsophagus occupies but twenty pages, and is altogether meager and imperfect: it is scarcely even a complete catalogue of the diseases of those parts. The section on the diseases of the peritoneum is more and better filled, but contains, we think, nothing which is not pretty generally known, or has not been already published in works that are commonly read. That on diseases of the stomach is altogether good, though here also we miss several things, and among them that which should have formed an important part, namely, the description of the various appearances of vascularity of the mucous membrane and the import of each.

*Inflammations of the stomach.* Rokitansky describes four varieties of this disease—the catarrhal and chronic, the croupous or plastic, the inflammation of the submucous tissue, and that which is due to the action of poisons. The signs ascribed to the first and last are well known, though indeed it is not always an easy matter in practice to determine when those of the former are present. The second, which is that commonly called idiopathic acute gastritis, never occurs, he says, as a primary disease, except in the flocculent aphthous exudations observed in children ; an opinion which is seldom questioned except by those who seldom make post-mortem examinations. But it is sometimes the result of a degeneration of the process of an exanthema, especially of typhus and variola, (of which more presently,) or of the passage of pus into the blood, especially in puerperal uterine phlebitis :

"The third variety, or submucous gastritis, occurs not rarely as a secondary process analogous to the metastases of certain acute specific dyscrasies, and is comparable with pseudo-erysipelas. . . . . . . One finds the wall of the stomach thickened, the submucous membrane turgid with pus, rotten and easily torn, the mucous membrane over it reddened, and in some places tense. In these places it at last bursts, most usually over a depôt of pus, and leaves numerous irregular openings, from which the pus oozes, as through a sieve, upon the internal surface of the stomach." (p. 183.)

*Perforation of the stomach.* One of the most complete descriptions in the volume is that of the perforating ulcer of the stomach ; of which, however, Rokitansky's monograph in the Oesterreichische Jahrbucher, which has been repeatedly copied, is so well known that we may pass it by.

*Softening of the stomach.* Among softenings of the stomach he describes two varieties, both idiopathic affections, and both to be carefully distinguished from the effects of digestion after death. The one is the gelatinous softening of the stomach in children, of which the characters, as they are commonly described, are generally known ; the other an analogous change occurring in vascular instead of anemic stomachs, and presenting corresponding differences of colour. He mentions also a similar disease as producing destruction of the lower part, and especially of the left side, of the œsophagus ; and in a subsequent part of the volume speaks of a similar affection of the intestinal canal, especially of the cæcum.

All these, as well as the similar softening and destruction of the diaphragm and other adjacent organs to which they lead after perforating the stomach, are, our readers must be aware, ascribed in England to the action of the digestive fluid continuing after death and decomposing the tissues with which it comes in contact. Evidence more than sufficient for this view may be found in the works of Hunter, Burns, and Carswell ; and we regret to find so experienced an observer as Rokitansky following the French (we had nearly said the anti-English) pathologists in the opposite opinion. Even his own observations, when fairly considered, are favorable to our view. He says :

"It is an important fact that softenings exactly similar to those just described may take place after death, as the results of a dead chemical process, in what is called the self-digestion of the stomach. The discrimination whether this or the morbid softening have taken place is, in many cases, very difficult; nay, unless respect be paid to the state of disease which has preceded death, and to the mode of dying, is even impossible. The following circumstances, however, may be diagnostic of the softening after death: 1. The absence of all phenomena indicative of a softening of the stomach, or of the morbid processes inducing it, during life. 2. Sudden natural or violent death, without preceding disease, during the process of digestion—the stomach being full of chyme. 3. Limitation of the softening to the mucous membrane, and especially to its projecting folds; together with, lastly, an extension of it beyond the ordinary limits of the morbid softening, and its existence chiefly at those parts on which the greatest quantity of the contents of the stomach has rested." (p. 200.)

Now these characters are, for diagnosis, valueless; they may, and often do, exist alike in the cases which Rokitansky regards as the results of disease and those which he admits to be the effects of digestion. For the first of them, there is no known reason why a stomach which has been functionally or even in some measure visibly diseased, should not be softened and digested after death as well as a healthy one. Gastric disturbance may therefore have existed without having been due to this complete disorganization of a part of the stomach which is found after death. For the second, we can only say that these are not the most common circumstances in which digestion of the stomach occurs; on the contrary, in the majority of persons dying suddenly it does not take place at all. As to the third sign, the limitation when it exists is only accidental, depending on the stomach being contracted; its absence, therefore, is no indication that the general softening is due, not to digestion, but to disease. And, for the last, the locality here ascribed to the digestive softening is exactly the same as that in which the supposed softening from disease is found, namely, some part of the cardiac half of the stomach.

It is clear, then, that Rokitansky cannot assign one distinctive character between the softening supposed to be from disease and that admitted to be from digestion. This is good evidence that they are identical; and the proof that both are due to digestion is completed by these facts—that the characters of the softening are not similar to those produced by any disease in analogous tissues—that they are not associated with any of those other changes which should accompany the progress of such extensive disorganization—that exactly the same changes may be artificially produced by the action of the digestive fluid on a dead stomach or other organ—that they have no corresponding symptoms during life—and that they frequently occur in the stomach of those (both men and animals) who have died during perfect health. It seemed necessary to enter thus at length into the question; for we shall probably ere long find this, as many other errors in continental pathology have been, imported and made popular in England.

After some minute enumerations and brief descriptions of the alterations in the size, form, and arrangement of the intestines, the reading of which was much like the perusal of a long index, we come to the account of their structural alterations—a section full of important subjects, to most of which Rokitansky has done ample justice.

*Inflammation of the intestinal mucous membrane.* He divides this into four classes—the catarrhal or erythematous, the croupous exudative or plastic, the typhous, and the dysenteric.

The type of the first, or *catarrhal inflammation* is the common diarrhœa from cold, mechanical or chemical irritation, venous congestion, &c. It may be acute or chronic; and in the latter case it may give rise to several well-known, though not generally appreciated morbid alterations, of which he enumerates as the chief, "the brown or slate-gray, or blueish-black discoloration of the mucous membrane," its increased thickness and general volume, the hypertrophy of the submucous and muscular coats, and "the profuse secretion of a turbid grayish-white, or a transparent, jelly-like, ropy mucus." But in more advanced cases the chronic catarrhal inflammation passes into ulceration, especially when acute attacks are frequently grafted upon it; and now—

"The mucous membrane is changed into a granulated tissue, which is of an intense red colour, easily torn, and on whose surface, as well as in its interior, suppuration ensues. From the surface the suppuration spreads in depth; in the interior it appears as a depôt of pus, which bursts inwards; in either case there is a loss of substance, which quickly or slowly increases, forming ulcers with swollen, irregular, indented, and undermined edges, and granulating bases which extend into the submucous tissue or even into the hypertrophied muscular coat, and burrow in it in the form of excavated passages, around which the mucous membrane is in the gray or slate-coloured or black state just described, or is in a condition of blenorrhœa, or not unfrequently presents polypous growths." (p. 230.)

An analogous form of disease is the *inflammation and ulceration of the follicles of the large intestines,* which occurs in protracted diarrhœa and lientery; and with which, as well as with that just described, we are better acquainted in England as the state produced by dysentery. In this disease, in addition to the changes detailed above, the follicles are found either merely enlarged and projecting in round or conical knots on the surface of the intestine, or containing small quantities of pus; or when the pus has been discharged, are replaced by small, round ulcers, with pale, or ash-coloured, or livid edges, and with dull-white or sometimes ecchymosed bases, formed of cellular tissue. These, however, enlarging and coalescing, soon produce an appearance nearly similar to that of the ordinary diffused catarrhal inflammation.

On the *typhous process* in the intestinal canal Rokitansky dwells at considerable length, and his remarks merit close attention. Of the typhous process in general he says (and this may express in a brief form his theory of it,) that, anatomically, it is characterized by the deposition of a peculiar product which passes through peculiar metamorphoses. This product (the typhous matter or structure—*typhus-gebilde*,) has, in its origin, and still more in its metamorphoses, the greatest analogy with the cancerous matter, and especially with that of the medullary cancer. The situation in which it is deposited varies according to the special relation of the general process to certain organic structures; but its most usual primary seat is in the mucous membranes (especially that of the small intestines,) and the lymphatic glands. The local typhous process is an inflammation; not, however a genuine inflammation, such as one supposes to depend on a phlogistic state of the blood, but a typhous inflammation, corresponding to and consequent on the peculiar morbid condition of the blood. The existence of such an inflammation in the small intestines in typhus is, in Germany, constant; but its development may be arrested, and therefore cases occur in which it seems to be absent, and in which the whole process seems to go on in the blood.

This brief summary of Rokitansky's pathology of typhus is necessary for the appreciation of his account of its morbid anatomy. He has probably carried his notion of the analogy between the materials of cancer and of typhus too far; but this may be discussed when he has published his first volume, in which these general questions will be treated. At present we proceed to the local morbid anatomy of the disease.

He divides the typhous process in the mucous membranes of the small intestines into the four stages of congestion; the deposition or infiltration of the typhous material in its crude state; its loosening, softening, and ejection; and the proper typhous intestinal ulcer. In the first stage there is venous congestion, swelling, and a peculiar succulency of the mucous membrane, especially of its lower part; it has a dull gray colour, and is covered by a thick layer of dirty yellow, jelly-like mucus.

In the second stage these signs are usually concentrated about the Peyer's patches and a few of the solitary glands, which are elevated by the deposition of the typhous material into the well-known *plaques.* This material is a more or less compact, pale, reddish, fibro-lardaceous (*faserig-speckige*), friable mass, which is sometimes traversed by bloody streaks. It very rarely extends beyond the glandular apparatus, and on close examination is found to be deposited in the Peyer's and solitary sacculi, below the mucous membrane, and in the submucous tissue; in such a manner that the layer of the latter, which is next the muscular coat, is unaffected. The mesenteric glands in this stage are much swollen, vascular, and firm.

When the third stage sets in the congestion is again increased, the mucous membrane and especially the villi swell up, and, on pressure, give out a grayish-white, flocculent fluid:

"But the most remarkable alteration takes place in the typhous patches (*plaques*) and in the mesenteric glands; they become loosened. The patches swell still more, and when the process does not set in and go on in them uniformly they acquire an uneven, nodulated surface. The substance deposited in them is changed into a grayish medullary material, and undergoes one of two changes: either it is converted, together with the mucous membrane covering it, into a closely adherent, dirty yellow or brown slough, infiltrated with the feculent contents of the intestines, which then shrivels up, gradually separates from the margins around it, becomes rotten, splits in different directions, breaks off from the deepest layer of the submucous tissue, and in this way is at once or piecemeal discharged; or else it degenerates (and this with peculiar intensity in certain epidemics) into a loose, vascular, blueish-red, fungous tissue, permeated by streaky extravasations of blood, or infiltrated with blood, which is the source of profuse intestinal hemorrhages, and is cast off piecemeal without precedent sloughing. This metamorphosis, which goes on in the solitary as well as in the Peyer's glands, though later and more slowly, sometimes affects the whole patch almost uniformly, but sometimes only particular portions or single follicles, while the rest of the patch undergoes a retrograde metamorphosis by absorption, collapses, and leaves behind it a flaccid, succulent, wrinkled swelling of the plexus of glands.......... The mesenteric glands, which in general are always behind the intestine in their typhous metamorphoses, attain in this third stage their extreme size. They become as large as pigeons' eggs, and in the neighbourhood of the cæcum as large as hens' eggs, and form a knotted cord, extending obliquely from the end of the ileum to the lumbar plexus. They are blueish or brownish-red, very vascular, and often are the seats of extravasations of blood." (pp. 240-2.)

This stage completed, the proper typhous ulcer remains, which is distinguished by its elliptical, round, or irregular form, (according as the whole of a patch, or a few of its glands, or a solitary gland, or irregular portions have sloughed;) its position opposite the attachment of the mesentery, and with its long axis parallel to that of the intestine; its livid or gray, loose and free, elevated and overhanging margin; its base formed by a delicate layer of the deepest submucous tissue; and its chief seat nearest the ileo-cæcal valve. Such ulcers may heal; and their cicatrices have this singular character, that they never produce any narrowing of the intestinal canal. The mesenteric glands at the same time shrink to their natural volume, or below it.

Among the alterations of other organs in typhus, Rokitansky mentions a flaccidity and paleness or dirty redness of the heart; but that softness which is described by Dr. Stokes does not occur among his patients. Since 1824 he has often observed that,

"In the first stages of typhus the ganglia of the solar and superior mesenteric plexuses are in a state of turgescence with a blueish or grayish-red colour, and a looseness of texture; and that in the stage of ulceration and subsequently, they are collapsed, pale, flaccid, and not unfrequently in a remarkable degree withered, leathery, and tough, and of a dirty white or ash-gray colour." (p. 247.)

But these are only the general features of the local typhous process. It presents, in certain cases, remarkable anomalies both of *quantity* and of *quality*, as Rokitansky distinguishes them. In the anomalies of the former kind there may be only a diffuse congestion of the mucous membrane, or an imperfect development of the patches, with but a slight plasticity of the morbid product; or the latter may be absorbed, producing the "plaques à surface reticulée" of Chomel; or its metamorphosis may make slow progress. Or, when the local process is intense, there may be severe congestion of the mucous membrane, extending even to the peritoneum, and producing peritonitis, with unusual turgescence of the morbid product, and effusion of blood in the coats of the intestine; or there may be exuberant fungous growths or destructive hemorrhage; or, in other cases, the morbid product may spread far beyond its ordinary bounds.

Of the anomalies *in quality*, the most remarkable are those in which the cicatrization of the ulcers is much retarded (which may be due to a variety of causes), and those in which the ulcers go on to perforation, a result which, Rokitansky thinks, is brought about by a process quite different from that of the previous and proper typhous ulceration. The latter is the consequence of the metamorphosis of the peculiar morbid product of typhus which is never deposited so deeply as the muscular coat. That coat, therefore, is the proper boundary of the typhous ulceration, and when the ulceration spreads more deeply, the tissue attacked by it does not first become the seat of typhous deposit. And in this, the progress of the typhous is distinguished from that of the tuberculous, ulcer of the intestines, which makes progress, both in breadth and in depth, by the softening and ulceration of secondary deposits of infiltrated or granular tubercle in its margins and base. The progress of the latter to perforation is tuberculous throughout; that of the former is typhous only at its commencement.

Again, there are cases of qualitative anomaly in the typhous process, in which the progress of the affection of the mesenteric glands is much more acute than usual, producing peritonitis, or effusions of blood into the peritoneum, or fungous degeneration and breaking out of the substance of the glands.

Lastly, Rokitansky describes briefly what he calls the "secondary typhous processes," some of which he names *genuine*, others *degenerate*. Those of the former class comprise the repeated eruption of the disease in the small intestines, its appearance in the colon and stomach, the air-passages, the pharynx, the urinary bladder, serous membranes, and various organs, all of which he believes are, in these cases, the seat, not of accidental ordinary inflammation, but of the deposition of typhous matter, which passes through its usual changes. The *degenerate* class of secondary typhous processes includes those examples in which, instead of the usual metamorphoses of typhous matter, the morbid process imitates that of a simple, non-specific disease, assuming the characters of a fibrinous or croupous inflammation, of gangrene, suppuration, or purulent diathesis; "all of which," says Rokitansky, "are based on a corresponding degeneration of the typhus-process in the blood." (p. 254.)

Such is Rokitansky's account of typhus; of which, as he proposes to develop it further in another volume, we will merely say that its foundation, taken only as an hypothesis, may be amply sufficient for the explanation of all the structural phenomena of the disease; but that, as a statement professing to be fact, it needs further palpable and visible evidence. Especially, we want at present the proof of the existence of this typhus-material as something possessing an elementary structure, or composition, different from that of ordinary exudative products. Its metamorphoses are peculiar, but so are those of many substances which as yet we regard as merely fibrinous. There is great probability of truth in Rokitansky's view, but we repeat that perceptible evidence is wanting. Moreover, if it be admitted, we think that the typhous bears more analogy to several other peculiar processes than to that of encephaloid cancer; surely, for example, it is much more like that of the exanthemata.

From the typhous disease of the intestines, Rokitansky proceeds to the *Tuberculous* and *Carcinomatous*. His descriptions of both are complete and good; and under that of the latter he introduces what seems a favorite "theory of ileus from scirrhous degeneration of the intestine," which, at least in part, is similar to that of Dr. Abercromby, in which the retention is ascribed not to spasm but to paralysis, not to the contracted but to the dilated portion of the canal.

"The cancerous portion of the intestine is, quite independent of the degree of stricture, in a completely passive state, in consequence of the firm, morbid matter, deposited in the submucous cellular tissue, and, still more, through the degeneration of its muscular coat. The onward motion of the intestinal contents through it is therefore effected by the muscular energy of the part of the canal above it; and this energy will be the more intense the more considerable the stricture, the greater the extent (in length) of the degeneration (although there be no stricture), and the greater the mass to be moved on. In all cases the intestinal contents will frequently stagnate in the canal above the diseased portion, and there they will accumulate, and distend it. Now this distension produces either (when it rapidly passes beyond a certain proportion) immediate paralysis of the tube, or it produces reaction, by which as much as possible of the contents is repeatedly forced through the diseased part; hence follows hypertrophy, especially of the muscular coat, and after this, when the accumulation and distension increasing gain the ascendancy, gradual exhaustion and paralysis. And this paralysed portion of the intestinal tube has in it the proximate cause of the ileus which soon ensues. As soon as the accumulation of the intestinal contents, continually passing into it, has become so great as to reach to that part of the canal above it which is still capable of contraction, this endeavours to force them on. But its most energetic action has little effect, since it has to overcome, not only the masses accumulated in the paralysed portion of the canal, but also, beyond them, a stricture. Under these circumstances the peristaltic motion becomes antiperistaltic, the intestinal contents are carried back to the stomach, and thence are discharged by vomiting." (p. 282.)

It is not difficult to apply this most ingenious and probable explanation of ileus from stricture to all the varieties of the disease, whether the paralysis be primary or secondary.

*Affections of the liver and gall-bladder.* After this, Rokitansky describes the diseases, or their varieties, peculiar to each division of the intestinal canal, and next the anatomy of the diseases of the liver, a subject of which he, like all who know anything about it, confesses the extreme difficulty. His description of the various appearances which they present is most complete and accurate; if we may so speak, his portraits are drawn to the very life; but still they are mere descriptions, of which neither an abstract nor a generalization can be made; and we must pass them over with no more than the assertion of our belief that there is not a man in Europe who will not be the wiser for having read them.

Under "Diseases of the Gall-bladder," the fact is pointed out that "in cases of obstruction of the ductus choledochus the dilatation gradually extends up the whole biliary apparatus, but the gall-bladder in such cases of even very remarkable dilatations, does not commonly present a proportional degree of distension, because the cystic duct, opening at an acute angle, is compressed by the dilated ductus choledochus" (p. 361); a circumstance of no small importance in the security which it affords against rupture of the gall-bladder.

He shows also how, after complete obliteration of the cystic duct, the gall-bladder acquires all the characters of a serous membrane, and becomes liable to the same diseases. "Especially, inflammations are not rare, which produce the most varied exudations, and lead to as different results. Among the latter, a shrinking of the gall-bladder, with diminution of its contents, and an inspissation of them into a fatty and calcareous pulp and concretion, and then the ossification of its walls are particularly to be remarked." (p. 363.)

*Diseases of the spleen and pancreas.* Under this head there is much accurately described, but little from which definite conclusions can be drawn. The descriptions of the diseases of the pancreas are more numerous and complete than those in any other work, but still scanty, and seemingly unimportant. These, therefore, with which the section on the digestive apparatus is terminated, we pass by.

*Affections of the urinary organs.* In the next chief section, he treats of diseases of the urinary apparatus, and again exhibits, in the midst of some obscurity of explanation, that remarkable power of recognizing and portraying differences and resemblances of mere appearance, which is the most striking feature of the work. From this section, since the division on the diseases of the kidneys is the only one presenting much novelty, we shall make a full, though but a partial abstract.

After passing briefly, in the plan adopted throughout the volume, over the anormal defects and excesses of development of the kidney, its deviations in size, in form, in position, and in consistence, and its solutions of continuity, he comes to that which here, as in other cases, constitutes the chief division,—the diseases of its texture. In these he places hyperemia and apoplexy, inflammation, Bright's disease, and morbid deposits. His account of the third of these is most worthy of notice; for though very complete, it is, we believe, as far as morbid anatomy is concerned, very true.

*Bright's Disease.* "Bright's disease appears in very different forms, which have relation partly to the degree and course of the disease, partly to its stage; and the first two of these circumstances are essentially connected with the degree of local reaction in the parenchyma of the kidney, and with the degree of morbid affection of the blood. We shall first point out the different modes of appearance of the disease as so many forms of it, then illustrate their combinations, their course, and their passage from one to the other in stages and degrees, and lastly, enter upon a general consideration of the disease." (p. 412.)

In this view Rokitansky arranges all the forms of this disease under eight varieties, of which we may say beforehand, that the first is peculiar, the next four are stages or modifications of the same general process, the sixth and seventh are peculiar, but related, and the eighth is a peculiar form, dependent on a peculiarity of the general diathesis.

"*1st Form.* The kidney appears enlarged, swollen, and heavy: the cortical substance is almost uniformly infiltrated by a dirty, reddish-brown, turbid fluid, on which, as a ground colour, the blood-vessels present a darker, sprinkled, or streaky redness. Other similar red spots are produced by effusions of blood in the tissue. The pyramids present the same dark discoloration, with dirty, streaky redness. The whole tissue, but especially the cortical substance, is uncommonly brittle, and easily torn; and on its torn or cut surface a reddish-brown, thin, very finely flocculent, turbid, bloody, and somewhat sticky fluid oozes out. The whole organ is turgid and flaccid. The capsule is injected, infiltrated by the dirty, bloody fluid, and easily separated; the mucous membrane of the pelvis, &c. is reddened and loosened, and contains muco-sanguineous, turbid, urinous fluid.

"*2d Form.* With an increase of size and weight as in the first, the tissue of the cortical substance appears either uniformly, or in certain undefined portions, infiltrated by a reddish, or yellowish-gray, or white, turbid, viscid fluid, from which it derives its characteristic colour, and which exhibits here and there an indistinct, punctiform, and linear arrangement. There is also a sprinkled or streaky redness, from injection or ecchymosis, which is the more striking the paler the infiltrated tissue is. Very often, the infiltration and bleaching of the tissue are complete in certain parts, while in others the hyperemia and ecchymosis preponderate; and this is the combination described by writers as a peculiar species, arising from partial anemia and hyperemia. The consistence of the organ is diminished, but less than in the first form. The capsule and mucous membrane are in the same state; and the pelvis, &c. contain a whey-like, turbid fluid, of a white colour, tending to yellowish or reddish. The pyramids commonly appear engorged, and therefore rather dark.

"*3d Form.* Considerable increase in size and weight, complete anemia of the cortical substance, which is interrupted only by scattered stars, knots, and streaks of blood-vessels. The cortical substance is increased between five and nine lines in diameter; its surface is smooth and slightly glistening, it is turgid, friable, and filled by a great quantity of a milky, turbid, whitish or yellowish fluid. Especially in the superficial layers, but in the deep ones also, it appears to consist of white or yellowish-white looser granules (Bright's granulations), varying in size between those of poppy-seeds and pins' heads; and towards the pyramids there are lines of the same substance. The pyramids are often compressed and separated from one another by the enlargement of the substance between them; and by the increase of that between their tubules, these are separated so that the bases of the papillæ seem fibrous or tufted. The capsule is very easily separated, swollen, and opaque; the mucous membrane reddened; the pelvis, &c. filled by a milky, turbid, viscid fluid.

"*4th Form.* Very considerable increase of size and weight; very considerable loosening of texture; the cortical substance is uncommonly turgid, and here and there feels almost fluctuating; its tissue is completely anemic, very easily torn, and filled by an unusual quantity of a milky-white, or yellowish juice. The granulations surpass the size of millet-seed; they are as large as hemp-seed; and, since this is especially the case in the peripheral layer, they project on the surface of the organ, and give it a glandular aspect. Sometimes a tumultuous enlargement of this kind affects certain portions of the kidney, and then an aggregate of these granulations projects, cauliflower-like, on the surface, so that the kidney becomes misshapen by unevenness and knot-like elevations. The granulations are very soft, and tear and break up under the slightest pressure of the finger. The capsule is almost loose; the cones are pale rose-red, or scarcely discernible; the pelvis, &c. are reddened, and contain a thick, creamy fluid.

"*5th Form.* The kidneys are large, or of their normal size, or are more or less considerably diminished; their surface is granulated or glandular, or, together with glandular prominent portions, there are irregular parts deepened in fossæ, sunk-in and cicatrix-like. The cortical substance is coarsely granular, loose, very vascular, and full of blood, and the vessels are varicose; or, when the decrease of the size of the organ is great, the cortical substance is pale yellowish, very deficient in vessels and blood, hard, tough like leather, and for the most part of a dense, cellulo-fibrous texture; while here and there in the sunken fossæ on the surface there is a similar tissue, of a whitish or ash-gray colour. Moreover, one often sees in the cortical substance sacculi of various size, and full of different fluids. When the kidney is large the capsule is loosely, when small very closely, connected. In the latter case it is thickened, and the fat around it is condensed. The cones are small, dense, and for the most part dirty-brown. The calyces and pelvis somewhat shrivelled.

"*6th Form.* With an unimportant increase of size and weight the cortical substance appears, in certain indefinite situations, either uniformly pale-reddish, or else (especially on close examination) of a mixed and confused pale red and whitish, or yellowish faint hue; it is infiltrated by a consistent substance like a thick cream, or coagulated albumen, and exhibits not only no loosening of its texture, but either its normal consistence or a much greater toughness. The capsule is slightly loosened; the pyramids, calyces, &c. are healthy.

"*7th Form.* For the most part an unimportant enlargement, sometimes a partial shrinking; increase of density and consistence. The cortical substance, in certain undefined and sometimes large portions, is pale, and of a dull-white colour, from a coagulated albuminous or apparently fibrinous substance, in which the renal tissue is altogether suppressed. The kidney at these parts is swollen by the morbid substance deposited in it, or else it appears shrunken, from the substance having acquired a cartilaginous aspect and resistance. One or more of the cones sometimes exhibit a similar metamorphosis. The capsule is thickened, and firmly adherent to the diseased portions; the pelvis and calyces are swollen.

"*8th Form.* The kidney is seldom importantly, but usually somewhat, enlarged, and, though it be of its normal size, is always remarkably firm. With a dirty-reddish or yellow-leather colour, the cortical substance appears of a waxen lustre, uncommonly hard and brittle, and is infiltrated by an albuminofibrous substance. Sometimes one sees a whitish, flocculent material, in the form of finely granular points and lines deposited in the tissue, from which the surface as well as the section acquires a marbled aspect." (pp. 413-17.)

Such are the forms of this important disease which Rokitansky believes must, in a general view, be enumerated. It was the more important to make this long extract, because at this time, when minute researches are being made into the subject, it is desirable that the general aspect of the morbid changes, in all their varieties, should be well known. It is absurd to talk of one microscopic character in a diseased structure which has at least eight gross forms; or to suppose that the finding of one kind of minute change will explain the pathology of this disease, more than the finding of pus-globules explains all the varieties of suppuration or ulceration.

Of these eight varieties, Rokitansky continues, the first seven are proved, by the phenomena observed during life in connexion with them, to belong to the Bright's disease. The first is extremely rare: the changes described are found in cases which have run a most acute course, destroying life, perhaps, in fourteen days. The second, third, fourth, and fifth, constitute the genuine Bright's disease, and Christison's Granular Degeneration,—a chronic, though often aggravated and accelerated affection, in which the first three of these forms are produced in successive stages, in either of which death may ensue, and in which the fifth form is the last member in the series, and indicates a retrograde process in the damaged tissue, *a secondary atrophy.* These four forms, or any two of them, moreover, may be found together, in one or both kidneys; and the disease in the cortical generally takes the lead of that in the deeper substance. The sixth and seventh are genuine chronic forms, the latter being the end of the metamorphosis, the product of the diseased process becoming condensed and organized, and shrinking up. The eighth form is also always chronic, occurring in inveterate scrofula, rickets, &c., and especially in the cachexia of syphilis and mercurial disease, and usually coinciding with infiltrations of similar substances in other organs. The essence of the disease is inflammatory:

"A process of inflammation, after hyperemia, deposits in the stage of *stasis,* a product which is not only distinguished by its peculiar nature, but which also, in the genuine cases, by its excessive accumulation, alters in a peculiar manner the aspect and the texture of the kidney. It is for the most part chronic, with occasional exacerbations; but sometimes is acute, and it is in these important cases, in which, in consequence of the rapid process of exudation, the product mixed with a great deal of serum, is not yet coagulated into the milky or creamy substance characteristic of Bright's disease, and is for the most part coloured by blood, that one would be obliged to regard the condition as a very acute simple inflammation of the kidneys, if the general phenomena and the state of the urine did not set the peculiar nature of the disease beyond a doubt." (p. 419.)

He believes that the product is deposited in every part of the tissue external to the vessels, but especially in the corpora Malpighiana; (but this, we think, not from personal observation;) that its peculiarities depend on a peculiar morbid condition of the blood "consisting in an excess of albumen, perhaps based on a *depotentiation* of the fibrine;" that the albumen in the urine is due to its discharge with the excretion coincident with its deposit in the tissue, the dropsy to the attenuation of the blood. The disease may get well in any of its stages by resolution; but far more commonly it goes on to granulation, induration, and atrophy. But on all these questions the information is meager.

From the rest of this section we find only scattered observations worth noticing; but these are not a few:

"*The dilatations of the ureters* induced by obstruction and accumulation of urine, are often favoured by an inflammatory state of their mucous membrane paralysing their contractile external layer." (p. 436.)

"*Dilatation of the pelvis of the kidney,* in a very remarkable case which fell under our observation, was the consequence of the pressure exercised by a branch of the renal artery passing in an arch from the upper end of the hilus to a branch lower down, and crossing over the pelvis at its passage into the ureter." (p. 438.)

"*The occurrence of cysts in the pelvis* and ureter appeared to be frequent, when compared with their existence in or on other ducts, and therefore deserves remark. They are cysts varying in size from that of grains of sand to that of peas which are developed under the mucous membrane, either singly or in groups, which contain either a colourless or yellowish serous fluid, or with this a soft, gelatinous, or hard, amber-like, horny lump, of various size, and which, moreover, occasionally bursts, together with the mucous membrane, over them; so that concretions of the same kind are occasionally found in the urinary bladder. They were situated, in the two cases observed, chiefly in the ureters; in one of them in the pelvis and calyces also." (p. 442.)

"*Primary croupous (fibrinous or plastic) inflammation of the mucous membrane of the bladder* is indeed one of the rarest of appearances, but exudative processes in the form of secondary affections are not so rare as is commonly believed. They occur in the course of exanthematous diseases, especially of variola and scarlatina, in the course of typhus as the expression of an anomaly and degeneration of the proper process, in consequence of the passage of pus into the blood, and in company with exudative processes on other mucous membranes. . . . . . . . . .

"*The typhous process on the mucous membrane of the bladder* occurs, 1st, very rarely in a genuine form, that is, characterized by a product like that which it deposits, especially in the intestines and mesenteric glands; 2d, very frequently degenerated into an exudative process, particularly in the form of scattered, insular, soft exudations; and, 3d, degenerated into an exudative process, of the nature of a slough. But the disease generally proves fatal too soon for us to have an opportunity of observing the progress of these products." (p. 456.)

"*Tubercle* very rarely occurs on the mucous membrane of the bladder. . . . . . . . . . . . . . . . It is usually associated with tubercle of the urethra and prostate. It always appears in the form of discrete granulation, which is deposited in and beneath the mucous membrane with more or less of reaction and development of vessels, which softens more or less rapidly, breaks through the mucous membrane within a circle of vessels, and forms a small, roundish ulcer. Secondary tuberculous deposits and secondary enlargements of the tuberculous ulcer occur but very rarely." (p. 458.)

The section is concluded by a brief account of the anormal characters of the urine, and of the deposits and concretions formed in it.

AFFECTIONS OF THE SEXUAL ORGANS. The section on the diseases of the male organs, like all those on affections in the province of surgery, is very incomplete. The only passage worth quoting is on

"*Inflammation of the vesiculæ seminales.* One has not unfrequently occasion to observe in the dead body a *chronic catarrh* and its consequences, consisting in swelling with relaxation of the mucous membrane, secretion of a grayish or yellowish puriform mucus, dilatation and, at last, thickening of the walls of the seminal vesicles. In rare cases one finds, after suppuration of the mucous membrane, that the affected parts are covered by a whitish or ash-gray latticework of a fibro-cellular texture, their walls very considerably thickened and brawny, their canal narrowed and obliterated. With equal rarity this inflammation degenerates into an ulcerative perforation of the seminal vesicles, the formation of depôts of pus in their cellulo-fibrous nidus, and even into destruction of a convolution and the laying open of two contiguous canals. The chronic catarrh occurs, especially in old age, in consequence of the mechanical hyperemia of the pelvic venous plexuses, ........ together with catarrhal states of the bladder after repeated gonorrhœa, excessive venery," &c. (p. 490.)

*Diseases of the female organs.* The section on this class of diseases, unlike the preceding, is very complete. One of its most interesting parts is that in which Rokitansky discusses at great length his favorite subject of the unicorn, bicorn, and bilocular malformations of the uterus; all of which he clearly shows to be dependent on different degrees of defect in the development and coalition of the two lateral halves of which the ultimately single organ is originally composed. The unicorn uterus he shows is one in which one half only of the organ has been developed; the bicorn, one in which both halves are developed, but have not coalesced so completely or so high up as they should; the bilocular, one in which the coalition is nearly complete externally but imperfect internally. But between these states there are various intermediate degrees, depending on the extent of coalition, and the inequality of the development of the two halves; for though both halves may be developed, yet one may be much less so than the other, and may appear as a mere rudiment of a uterine horn attached to the side or fundus of the more perfect half. Of this last case he mentions a very remarkable example:

"There is in our collection an example of pregnancy in a rudimental uterine horn, which proved fatal in the third month by rupture and effusion of blood into the peritoneal sac. The case had been regarded as one of Fallopian pregnancy, till a recent examination of it led me to another view................ The true uterus is a unicorn uterus of the left side, with a vaginal portion in which one may perceive signs of former gestations; from its extremity, which is arched towards the left, proceeds the left Fallopian tube. Into the convex right border of this uterus there sinks a flattened hollow cord, composed of uterine tissue, with tolerably thick walls, the cavity of which opens by a narrow aperture into the cavity of the true uterus. This cord is more than two inches long, and externally is enlarged into a sac, from the outer end of which there proceeds a (right) Fallopian tube, which has its ovary, and below it a round ligament. The sac contains a female fœtus, of three months, with its membranes,............. and is burst near the insertion of the umbilical cord. The left half of the uterus is twice as large as in the unimpregnated state, and thickly walled; its inner surface, as well as that of the canal opening into it, is lined by decidua, and its cervix is filled by a plug of jelly. The parts were taken from a girl twenty-four years old............ Four pints of blood had flowed from the ruptured sac into the abdomen." (p. 519.)

Cases of this kind prove the possibility, and even the probability of superfœtation occurring in the subjects of the malformation. And many of the cases which Rokitansky has observed confirm Meckel's observation, that women with uteri thus formed are peculiarly liable to die in gestation and after delivery; whether from defective power of the half uterus, or from the hindrance produced by the simultaneous enlargement of the unimpregnated portion, or by the oblique position of the part in which the fœtus lies, or from the defective supply of blood to the impregnated part, a large portion of that which is sent being appropriated by the enlarged unimpregnated part.

Under the organic affections of the uterus Rokitansky says, in explanation of—

"*Hydrometra, or uterine dropsy.* In consequence of inveterate uterine catarrh, strictures and closure of the cavity not unfrequently take place, and from these, when the catarrh continues, dilatations of its cavity and cervix. In the further course of a dilatation thus continuing, one sometimes observes in the uterus that alteration in its texture and function which occurs under similar circumstances and produces dropsy of other mucous canals and cavities, as of the gall-bladder already mentioned. The mucous membrane of the uterine cavity, stretched by the accumulating secretion, becomes gradually a thin serous membrane, which secretes a colourless, serous, albuminous, synovia-like fluid. The uterus is changed into a round, pretty-thickly walled, dropsical capsule, as large as an egg or a fist; a state which alone properly deserves the name of *hydrometra*............ The fluid may, through disease of the lining membrane, be variously changed, or may be partially discharged through the vagina, and accumulate again." (p. 536.)

Among the changes which the uterus may undergo in consequence of the development of fibrous tumours, he mentions as the most important the elongation produced by the growth of many tumours, and their being carried upwards out of the pelvis; and adds:

"The uterus thus becomes thinner: nay, in rare cases the atrophy is carried to such an extent that the uterus slowly suffers a solution of continuity; one portion of it remaining attached to the vagina, while the other is dragged upwards till the two are connected only by a cord of fibro-cellular tissue." (p. 547.)

A section is devoted to the *diseases of the impregnated uterus and of the puerperal state.* Those of the latter cannot be well described in an abstract; nor, except in its completeness, does his account of them present much that is novel. The following, however, is certainly not generally known:

"A very remarkable and, from its dangerous nature, a very important state, which may be recognized even in the dead body, is that of a paralysis of the portion of the uterus on which the placenta is implanted, while the part around it has undergone its normal changes. It presents a peculiar appearance. The situation of the connexion of the placenta is pressed in towards the cavity by the surrounding contracting tissue, so that it projects in the form of an arched tumour, while externally one observes on the corresponding part a shallow inversion of the uterine wall. One is easily induced by a deceptive resemblance to regard the paralysed section of the uterus as a fibrous polypus, and only an accurate examination of its tissue can afford a decided conclusion. The disease induces exhausting hemorrhages, which continue even for several weeks after delivery, and thus proves fatal. We have seen it twice—once after an abortion and once after delivery at the full term; and probably other cases are recorded." (p. 556.)

From the diseases of the Fallopian tubes, ovaries, and mammary glands, which close this section, Rokitansky proceeds to *diseases of the ovum and fœtus*, of all of which he gives a complete, though necessarily brief, sketch, and thus concludes the volume. In the course of our analysis of it we have said enough of its defects, and comparatively little of its merits. The best evidence of the latter is found in the length to which this abstract has been carried without passing beyond the bounds of what is novel or important. Nor would that fault have been committed though much more had been borrowed. No modern volume published on morbid anatomy contains half so many genuine facts as this: it is alone sufficient to place its author in the highest rank of European medical observers.

